# Both Pre- and Postsynaptic Activity of Nsf Prevents Degeneration of Hair-Cell Synapses

**DOI:** 10.1371/journal.pone.0027146

**Published:** 2011-11-03

**Authors:** Weike Mo, Teresa Nicolson

**Affiliations:** Howard Hughes Medical Institute, Oregon Hearing Research Center and Vollum Institute, Oregon Health and Science University, Portland, Oregon, United States of America; Virginia Commonwealth University Medical Center, United States of America

## Abstract

Vesicle fusion contributes to the maintenance of synapses in the nervous system by mediating synaptic transmission, release of neurotrophic factors, and trafficking of membrane receptors. N-ethylmaleimide-sensitive factor (NSF) is indispensible for dissociation of the SNARE-complex following vesicle fusion. Although NSF function has been characterized extensively in vitro, the in vivo role of NSF in vertebrate synaptogenesis is relatively unexplored. Zebrafish possess two *nsf* genes, *nsf* and *nsfb*. Here, we examine the function of either Nsf or Nsfb in the pre- and postsynaptic cells of the zebrafish lateral line organ and demonstrate that Nsf, but not Nsfb, is required for maintenance of afferent synapses in hair cells. In addition to peripheral defects in *nsf* mutants, neurodegeneration of glutamatergic synapses in the central nervous system also occurs in the absence of Nsf function. Expression of an *nsf* transgene in a null background indicates that stabilization of synapses requires Nsf function in both hair cells and afferent neurons. To identify potential targets of Nsf-mediated fusion, we examined the expression of genes implicated in stabilizing synapses and found that transcripts for multiple genes including *brain-derived neurotrophic factor (bdnf)* were significantly reduced in *nsf* mutants. With regard to trafficking of BDNF, we observed a striking accumulation of BDNF in the neurites of *nsf* mutant afferent neurons. In addition, injection of recombinant BDNF protein partially rescued the degeneration of afferent synapses in *nsf* mutants. These results establish a role for Nsf in the maintenance of synaptic contacts between hair cells and afferent neurons, mediated in part via the secretion of trophic signaling factors.

## Introduction

The fusion of vesicles at the presynaptic membrane is fundamental to synaptic transmission and neuronal function. Vesicle fusion is thought to involve the SNARE-complex, an evolutionarily conserved molecular machine that includes Rab GTPase, Munc18-like proteins, and SNAREs (soluble N-ethylmaleimide-sensitive factor (NSF) attachment receptors) as well as NSF, an AAA ATPase required for dissociation of SNARE complexes [1]. Defective SNARE-complex proteins have been shown to be associated with neurodegenerative disorders. For example, mouse models with mutations in the SNARE-complex and associated genes such as *Vti1a* and *Vti1b* [2], and *Munc18-1* [3] exhibit severe neurodegeneration. Botulinum neurotoxin C1, which cleaves SNAP25 and Syntaxin, also causes neurodegeneration [4], [5]. In addition, lethal mutations in *Drosophila dNSF1* have been shown to cause a reduction in synaptic branching of neuromuscular junctions [6]. These results indicate that components of the vesicle fusion machinery are also required for maintenance of synaptic contacts.

In this study, we examined the role of *nsf* genes in innervation of sensory hair cells of the zebrafish lateral line organ. This organ consists of superficial clusters of hair cells, called neuromasts, positioned along the head and trunk that detect water flow [7]. Neuromasts are innervated by anterior and posterior lateral line afferent neurons. Despite the extensive research on hair-cell neurotransmission, there are few reports on how SNARE-mediated release may influence formation and/or maintenance of hair-cell synapses. As in *Drosophila*, two *nsf* genes, *nsf* and *nsfb*, are present in zebrafish [8]. Mutant *nsf* zebrafish are paralyzed, possess enlarged melanophores, and have reduced clustering of sodium channels at the nodes of Ranvier due to myelination defects [9]. Mutant *nsfb* larvae, which were isolated in an insertional mutagenesis screen, display ubiquitous, nonspecific degeneration [10]. In this study, we characterized synaptogenesis in both mutants and our results indicate that *nsf*, but not *nsfb*, plays a role in maintenance of hair-cell synapses.

## Results

### Innervation in zebrafish nsf and nsfb mutants

**Figure 1:**
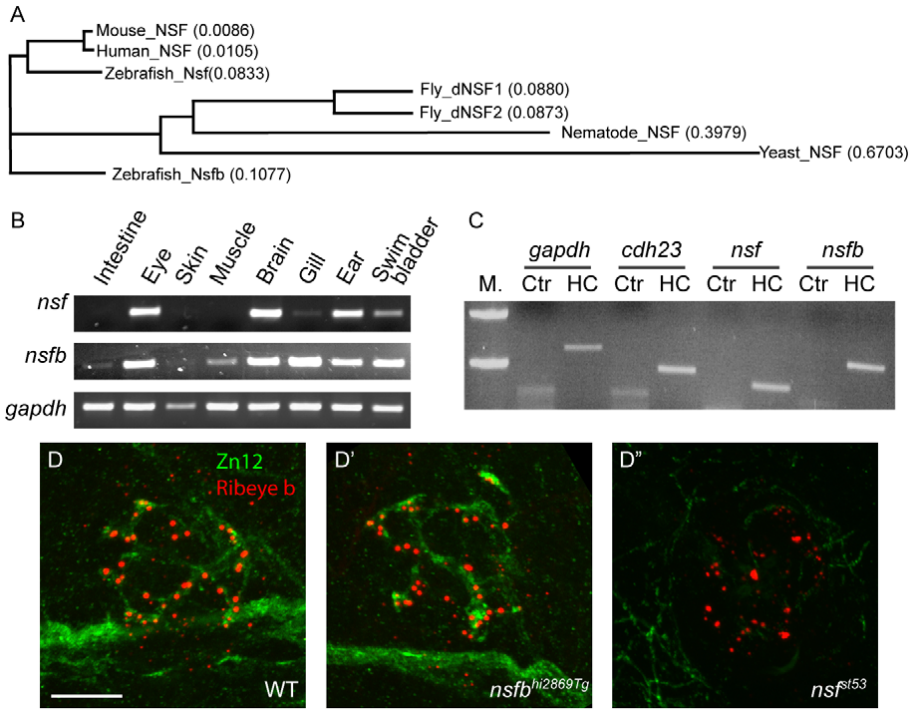
Expression of *nsf* and *nsfb* in zebrafish, and defective innervation of hair cells in *nsf* mutants. **A**, Protein sequences of NSF from yeast, nematode, fly, zebrafish, mouse and human were aligned using ClustalW to generate a phylogenic tree. The total amino acid substitutions of a specific protein are proportional to the length of each branch. The substitute rates of single amino acid were numbered in brackets after each protein. **B**, Detection of *nsf* and *nsfb* transcripts in adult zebrafish tissues by RT-PCR. **C**, RT-PCR of neuromasts isolated from 5 dpf larvae. *cdh23* was a positive control as a neuromast-specific gene. M. = Marker/DNA ladder, Ctr = Control with no reverse transcriptase, HC = Hair cell. **D**, Larvae (4 dpf) labeled with nerve fiber-specific Zn12 (green), and anti-Ribeye b antibodies (red). The merged images showed the innervation of the first lateral line neuromast (L1) by posterior lateral line neurons. Scale bar, 10 µm. The position of the specimen is dorsal up and anterior to the left, and each image is a projection of 10 optical sections (1 µm each). Scale bar is 10 µm.

The zebrafish genome contains two copies of *nsf*, *nsf* (also called *nsfa*) and *nsfb*. Nsf and Nsfb proteins share 83% identity and 91% similarity to each other. In the phylogenic tree, zebrafish Nsf is more similar to mammalian NSFs than Nsfb (Figure 1A). To determine the spatial expression of the two *nsf* orthologs in zebrafish, we examined the expression of *nsf* and *nsfb* transcripts in different tissues. An RT-PCR experiment was used to detect both *nsf* and *nsfb* mRNA in adult tissues. *nsf* mRNA was detected in mainly neuronal tissues, but not in non-neuronal tissues (Figure 1B). In contrast, *nsfb* was expressed in both neuronal and non-neuronal tissues (Figure 1B). To ascertain whether both genes were expressed in hair cells, we isolated single neuromasts from 5 dpf zebrafish larvae and performed RT-PCR [11]. We detected the presence of both *nsf* and *nsfb* transcripts in neuromasts (Figure 1C).

**Figure 2:**
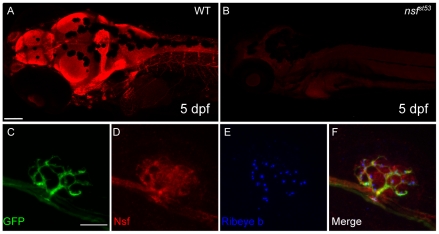
Expression of Nsf in the CNS and lateral line organ (5 dpf). **A**, An antibody against human NSF protein labels the larval nervous system (5 dpf). **B**, Labeling is absent in *nsf^st53^* mutants. Images are projections of 5 optical sections of 10 µm thickness. Scale bar, 100 µm. **C-F**, Confocal projections (10×1 µm sections) of anti-NSF (red) and Ribeye b (Blue) in *TgBAC(neurod:EGFP)nl1* transgenic fish. Nsf signal colocalized with anti-GFP (green) in the afferent nerve, and was also in Ribeye b-positive hair cells. Scale bar, 10 µm.

Because both *nsf* genes are expressed in neuromasts, we examined the contribution of both genes to ribbon-synapse formation in hair cells. We obtained *nsf^ st53^* and *nsfb^hi2869Tg^* mutant lines: the *nsf^ st53^* mutation results in a truncated protein before the second ATPase domain [9], whereas the *nsfb^hi2869Tg^* mutation results in a truncation of *nsfb* gene after the 18^th^ exon, also before the second ATPase domain [10]. The *nsf* and *nsfb* genes are on chromosome 3 and 12, respectively. Both mutants are paralyzed at 4 dpf. Whereas *nsf^st53^* mutants survive until 7 to 8 dpf, most *nsfb^hi2869Tg^* mutants die at 5 dpf. Therefore, we examined hair-cell innervation at 4 dpf, the latest stage possible for *nsfb^hi2869Tg^* mutants. In wild-type larvae, afferent nerve fibers form an elaborate pattern or web beneath neuromast hair cells [12], [13]. Typically, this highly branched structure is formed by two ganglion neurons, which make extensive contacts with the basal surfaces of the hair cells. The web of nerve fibers is more apparent when imaged using a top down view of the neuromast (Figure 1D and Figure 2C). Although the nerve fibers do not form distinctive boutons, the location of active zones can be visualized with antibodies against a ribbon-specific component, Ribeye b (Figure 1D and 2E) [13]. We compared innervation of neuromast hair cells in *nsf^st53^* and *nsfb^hi2869Tg^* mutants at 4 dpf, before extensive degeneration was visible in *nsfb^hi2869Tg^* mutants (Figure 1D′-D″) Although the overall number of neuromasts was reduced in *nsfb^hi2869Tg^* mutants, when hair cells were present, we observed innervation by the afferent nerve (n = 20 neuromasts, 5 larvae from 2 independent experiments) (Fig 1D′). Surprisingly, lateral line nerve fibers were mostly absent in *nsf^st53^* mutants at the same developmental stage (Figure 1D″). Together, these results suggest that Nsf, but not Nsfb, is required for stable afferent innervation of hair cells. Consequently, in the following experiments, we exclusively focused on the role of Nsf in the innervation of lateral-line hair cells.

### Degeneration of hair-cell afferent synapses in *nsf* mutants

To study the defects in afferent innervation in *nsf^st53^* mutants, we determined whether Nsf is expressed in both lateral line hair cells and afferent neurons. Using immunohistchemistry, we detected Nsf protein predominantly in the nervous system of 5 dpf larvae (Figure 2A), but not in *nsf^st53^* mutants, which have a stop codon positioned before the epitope of the NSF antibody (Figure 2B). To determine whether Nsf is present in afferent neurons, we used *TgBAC(neurod:EGFP)nl1* fish to mark afferent neurons and observed Nsf protein in GFP-positive lateral line nerves (Figure 2C-D). Co-labeling with Ribeye b antibody revealed that Nsf is also present in hair cells, consistent with our RT-PCR analysis (Figure 2E-F). Nsf was also detected in the cell bodies of the posterior lateral line ganglion (pLLG) and hair cells in the inner ear (data not shown). Taken together, these data suggest that Nsf function may be required in both pre- and post-synaptic cells for innervation.

**Figure 3:**
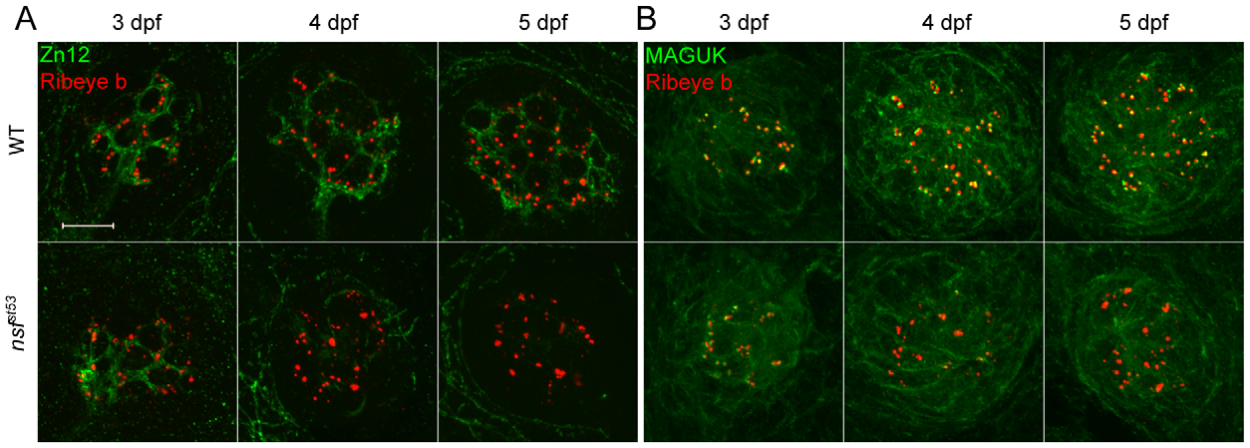
Degeneration of hair-cell synapses in *nsf^st53^* mutants during development. **A**, Antibodies against Zn12 (green) and Ribeye b (red) were used to label afferent fibers and hair-cell ribbons, respectively, in wild-type (WT) and *nsf^st53^* mutant larvae at 3, 4 and 5 dpf. **B**, Anti-Ribeye b (red) and MAGUK (green) labels the pre- and postsynaptic compartments respectively in neuromasts of wild-type and *nsf^st53^* mutants (3 to 5 dpf). Scale bar: 10 µm; z-projection of 10 confocal planes (1 µm each).

To determine if the lack of afferent innervation in *nsf^st53^* mutant hair cells is due to a failure to initiate synaptogenesis, or to maintain synaptic contacts, we examined afferent innervation of *nsf^st533^* mutant hair cells at different developmental stages using Zn12 as a marker. We found that afferent innervation of lateral line neuromasts was relatively normal at 3 dpf (Figure 3A, 5D). However, while the basket-like network of neurites was elaborated in wild-type embryos over days 4 and 5, *nsf^st53^* mutants exhibited a loss of hair-cell innervation (Figure 3A, 5D). The retraction of neurites was further confirmed by acetylated Tubulin antibody (data not shown). Consistent with these results, immunolabel of the postsynaptic marker Membrane-Associated Guanylate Kinases (MAGUK) was also reduced at later stages in *nsf^st53^* mutants (Figure 3B). These data suggest that Nsf function is not required to initiate hair-cell synaptogenesis in the lateral line organ, but instead is required for the maintenance of afferent innervation of hair cells.

**Figure 4:**
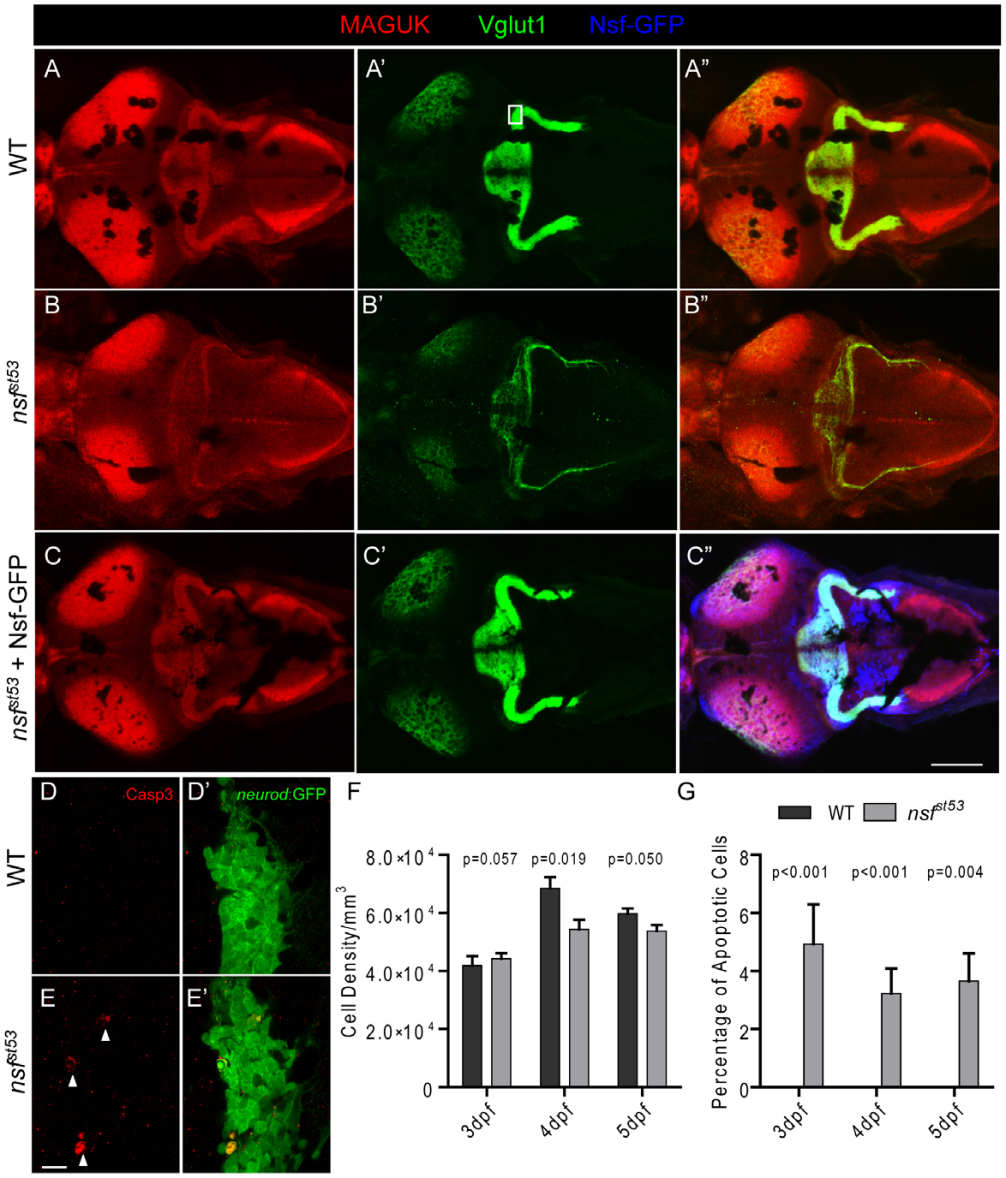
Reduction of glutamatergic synapses in the CNS of *nsf^st53^* mutants (5 dpf). Representative z-projections of the optic tectum and cerebellar region from a top view are shown. Wild-type (**A**), *nsf^st53^* mutant (**B**), and *nsf^st53^* mutant in *Tg(-5neurod:nsf-GFP*)vo1 background (**C**), which expresses Nsf-GFP (Blue) in various neurons, were labeled with Vglut1 and MAGUK antibody labeling. Scale bar: 100 µm; z-projection of 15 confocal planes (10 µm each). **D-E**′**,** Magnified views from the boxed region in panel **A**′. Activated Caspase 3 (Casp3) antibody was used to label apoptotic cells (arrow heads) in the cerebellar region, and GFP signals from *TgBAC(neurod:EGFP)nl1* fish were used to identify neurons, likely the Purkinje cells, in the cerebellum. **F**, Cell density (3 dpf, 41875.0±3287.4, n = 4; 4 dpf, 68500.0±3840.6, n = 5; 5 dpf, 59687.5±1856.3, n = 8 in wild-type and 3 dpf, 44285.7±1867.2, n = 7; 4 dpf, 54375.0±3264.7, n = 8; 5 dpf, 53750.0±2116.4, n = 6 in *nsf^st53^*) and (**G**) the rate of apoptosis in the cerebellum of wild-type (0% for 3 dpf to 5 dpf, n = 8) and *nsf^st53^* mutants (3 dpf, 4.3±1.3%; 4 dpf, 3.2±0.9%; 5 dpf, 3.1±1.0%, n = 8). P values compare *nsf^st53^* mutants to their wild-type siblings at the same developmental stage.

### Neurodegeneration in the CNS of *nsf* mutants

Because hair-cell ribbon synapses are highly specialized [14], we investigated whether Nsf activity is also required for the formation of conventional glutamatergic synapses. We labeled excitatory synapses with antibodies against Vesicular glutamate transporter 1 (Vglut1) and MAGUK, the pre- and postsynaptic markers of glutamatergic synapses in the CNS (Figure 4A-A″). In overviews of the larval brain, the labeling of Vglut1 and MAGUK was greatly reduced in *nsf^st53^* mutant larvae at 5 dpf (Figure 4B-B″), indicative of a reduction, and possibly degeneration of glutamatergic synapses in CNS. In a magnified view of the boxed region in Figure 4A′, Vglut1-labeled presynaptic terminals and postsynaptic densities stained by MAGUK antibody were mainly juxtaposed in both wild-type and mutant larvae (Figure S1). However, the Vglut1 and MAGUK label in this region of the cerebellum was highly variable even in wild-type larvae, preventing reliable quantification of the density or number of the synapses.

**Figure 5:**
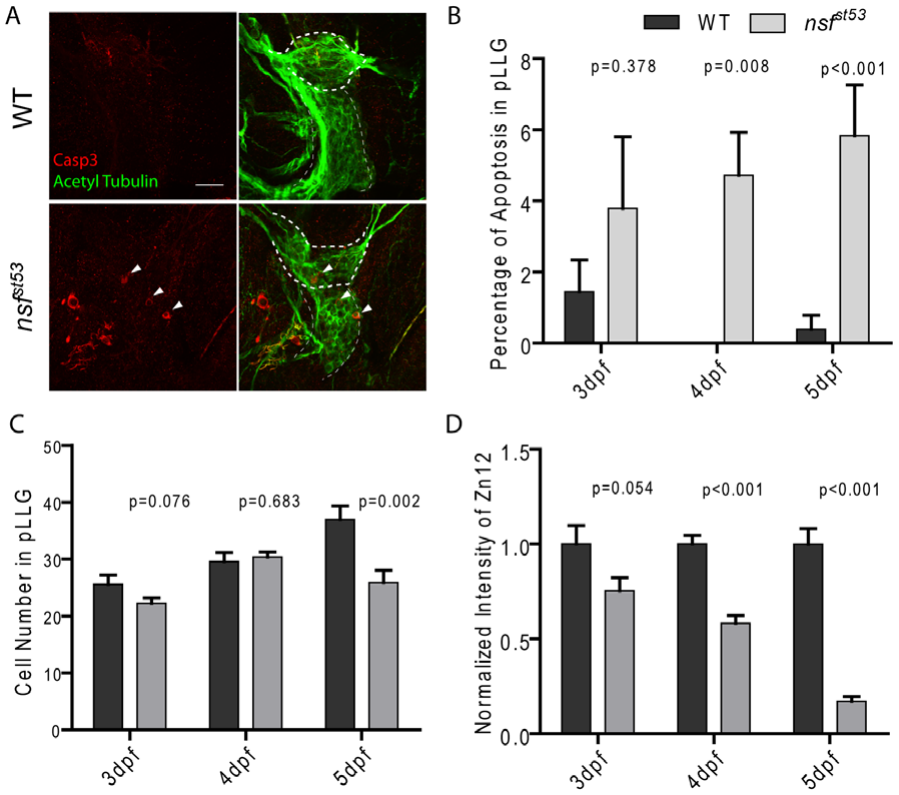
Apoptosis in *nsf^st53^* mutants (5 dpf). Representative z-projections of the pLLG (thick dashes) and vagal ganglion (thin dashes) are shown in (**A**). In WT and *nsf^st53^* mutants, apoptotic cells (arrow heads) were labeled by Casp3 antibody (red), and neurons were labeled by acetylated Tubulin antibody (green). Scale bar: 20 µm. Images shown in all panels are 6 z-stacks of 1.5 µm. **B**, The percentage of apoptosis in the pLLG of both wild-type and *nsf^st53^* mutant larvae (in wild-type the percentage apoptosis are 1.4 ±0.9, n = 5, 0.0 ±0.0, n = 8 and 0.4 ±0.4, n = 8 from 3 dpf to 5 dpf respectively; 4.8 ±2.0, n = 7, 4.7 ±1.2, n = 7, and 5.8 ±1.4, n = 7 in *nsf^st53^* mutants). **C**, Cell numbers of the pLLG in wild-type increased from 25.6 ±1.6, n = 5 at 3 dpf to 29.6 ±1.5, n = 5 at 4 dpf and reached 37.0 ±2.3, n = 8 at 5 dpf, while the numbers changed from 22.3 ±0.9, n = 8 to 30.4 ±0.8, n = 8 and then to 25.9 ±2.2, n = 7 in *nsf^st53^* mutants. **D**, The ratio of the intensity of Zn12 labeling (Figure 3A) of *nsf^st53^* to wild-type labeling declined from 3 dpf to 5 dpf (76.2 ±9.5, n = 6, 58.2 ±4.1, n = 5, and 17.2 ±2.4, n = 7).

To determine if we could rescue the degenerative phenotype in the CNS, we stably expressed Nsf-GFP in the CNS using a minimal *neurod* promoter, which drives expression in cranial nerves and other regions of the brain. We observed robust rescue of the reduction of both Vglut1 and MAGUK in *nsf^st53^* mutants in a *Tg(-5neurod:nsf-GFP)vo1* background. A representative image of the rescue is shown in Figure 4C-C″ (n = 10, from 3 independent experiments). These experiments suggest that expression of Nsf is sufficient to stabilize innervation within the brain, and that the C-terminally tagged version of Nsf is functional.

### Apoptosis in *nsf* mutants

To determine whether the retraction of the afferent nerve was due to cell death, we used an antibody against cleaved Caspase-3 to detect apoptotic neurons in 5 dpf larvae [15]. The overall morphology of the ganglion was normal in mutants, however, we observed a slight increase in activated Caspase-3 labeling in the pLLG of *nsf^st53^* mutants (Figure 5A-B). Labeling of activated Caspase-3 was not detectable in hair cells. Although the retraction of neurites in the *nsf^st53^* mutant occurred at every synapse at 5 dpf, apoptosis was detected in only a few cells within the pLLG at this stage. To determine if apoptosis caused the retraction of afferent nerves, we counted the number of neurons in the pLLG at different developmental stages, as well as quantifying the degeneration of afferent nerve fibers. We observed around 5% general apoptosis occurring between 3 dpf and 5 dpf in *nsf* null mutants (Figure 5B), with the number of cells in the pLLG in mutants being reduced by 30% at 5 dpf compared to their wild-type siblings (Figure 5C). During the same time interval, the reduction of innervation was much greater. From 3 dpf to 5 dpf, the afferent innervation of hair cells was reduced by more than 80% (Figure 5D). Because most pLLG neurons innervate a single neuromast [11], [16], it is unlikely that the cell death of pLLG neurons accounts for the total loss of innervation.

As seen in the pLLG, we also observe increased Caspase-3 labeling in the CNS, a sign of apoptosis in these cells (Figure 4D-E′). We also counted the number of apoptotic cells in the boxed region of Figure 4A′. Similar to the pLLG, the rate of apoptosis was low in the CNS and the cell density did not change drastically in the mutants (Figure 4F and 4G). Our data suggests that the decrease of Vglut1 and MAGUK labeling at 5 dpf is unlikely due to the loss of neurons, but rather that glutamatergic synapses degenerate in the *nsf^st53^* mutant.

### Pre- and postsynaptic function of Nsf

**Figure 6:**
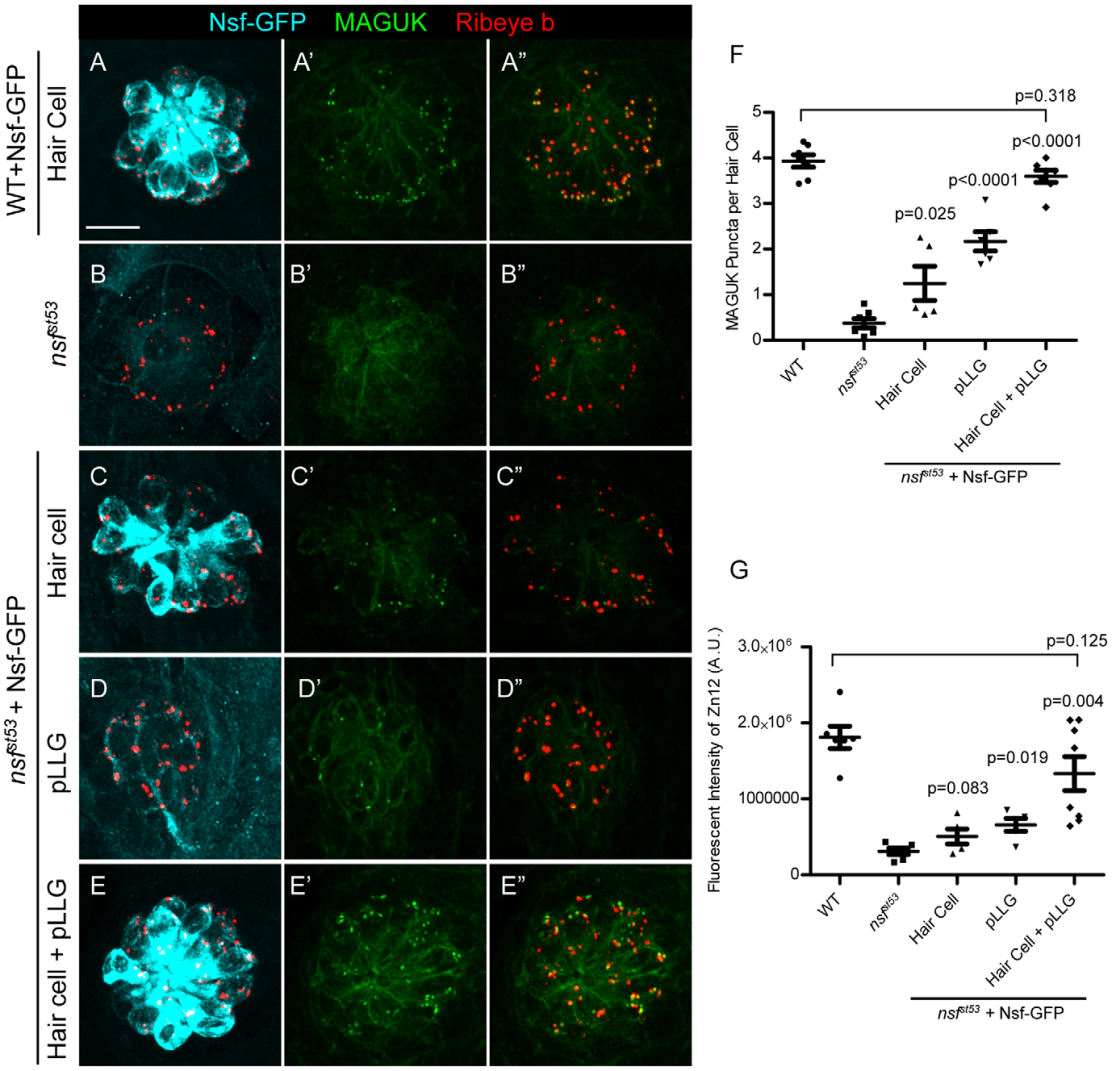
Nsf function is required in both pre- and postsynaptic cells to stabilize afferent innervation of hair cells. **A-E**″, Representative z-projections of a single neuromast from a top-down view (5 dpf). Wild-type (**A**), *nsf^st53^* mutants (**B**), and *nsf^st53^* mutants with Nsf-GFP expressed in hair cells (**C**), pLLG (**D**), or in both hair cells and pLLG (**E**) were labeled by antibodies against GFP (pseudo-colored light blue), MAGUK (green), and Ribeye b (red). Scale bar: 10 µm. **F**, The average number of MAGUK puncta per hair cell in wild-type (3.8 ±0.1, n = 7), *nsf^st53^* mutants (0.4±0.1, n = 7), and *nsf^st53^* mutants with hair-cell Nsf-GFP (1.2±0.4, n = 5), pLLG Nsf-GFP (2.2±0.2, n = 6), or both hair-cells and pLLG Nsf-GFP (3. 6±0.1, n = 7). **G**, Zebrafish larvae were stained with Zn12 and Ribeye b antibody. The fluorescent intensity (A.U.) of Zn12 was quantified in wild-type (1.813e6±147069, n = 6), *nsf^st53^* mutants (311769±43541, n = 6), and *nsf^st53^* mutants with Nsf-GFP rescued in hair cells (507787±97798, n = 5), pLLG (659674±85851, n = 5), or both hair cells and pLLG (1.335e6±223319, n = 8). The p-values were generated comparing the data from the *nsf^st53^* mutant to each transgenic mutant line.

Although the neurodegenerative phenotype of *nsf^st53^* mutants can be rescued by expression of Nsf-GFP in the CNS (Figure 4A-C″), it was not clear whether presynaptic Nsf originating from hair-cells would be sufficient to stabilize ribbon synapses. We sought to answer this question by targeting expression of Nsf-GFP to either hair cells, or afferent neurons of the pLLG. In addition to the minimal *neurod* promoter, we used another minimal 6kb promoter from the *myo6b* gene, which specifically expresses in hair cell [11]. We utilized the *Tg(-5neurod:nsf-GFP)vo1* for expression in the afferent neurons of the pLLG and generated a second line, *Tg(-6myo6b:nsf-GFP)*, to drive expression of Nsf-GFP in hair cells. We characterized the expression of Nsf-GFP in our stable transgenic lines using antibodies against both NSF and GFP (data not shown), and found three transgenic lines with distinct expression patterns: *Tg(-6myo6b:nsf-GFP)vo1* expressed Nsf-GFP only in hair cells; *Tg(-6myo6b:nsf-GFP)vo2* expressed Nsf-GFP in both hair cells and afferent neurons; and *Tg(-5neurod:nsf-GFP)vo1* used for the CNS experiments expressed Nsf-GFP in pLLG neurons, but not hair cells. Innervation of hair cells in mutants was analyzed in the three different transgenic backgrounds and compared to both wild-type and *nsf^st53^* mutant fish without Nsf-GFP (Figure 6). To evaluate and compare hair-cell innervation, we imaged the same neuromast (L1) in each specimen [17]. Expression of Nsf-GFP in either hair cells or afferent neurons in *nsf^st53^* mutants resulted in an increased number of MAGUK punctae (Figure 6C-D″, 6F). Although the number of MAGUK punctae was significantly increased in rescued mutants, the rescue was only partial in comparison to wild-type controls (Figure 6A-A″, 6F). Full rescue was only observed if Nsf-GFP was expressed in both pre- and postsynaptic compartments in the *Tg(-6myo6b:nsf-GFP)vo2* line (Figure 6E-E″, 6F). Quantification of the fluorescent signal obtained with the afferent fiber antibody Zn12 was consistent with the above results in each condition (Figure 6G).

The expression of the transgene in *Tg(-6myo6b:nsf-GFP)vo2* fish was higher than *Tg(-6myo6b:nsf-GFP)vo1,* but this may be partially due to the expression of Nsf-GFP in afferent nerve fibers, which cannot be separated from hair-cell GFP signals (Figure S2). To confirm that full rescue of hair-cell innervation was dependent on expression by both cell types rather than level of expression, we crossed *Tg(-6myo6b:nsf-GFP)vo1* and *Tg(-5neurod:nsf-GFP)vo1* into the *nsf^st53^* background. Double transgenic expression of Nsf-GFP yielded better rescue than either transgenic in mutant background alone, as quantified by Zn12 antibody labeling of afferent nerves (Figure S3). These results demonstrate that Nsf function is required in both hair cells and pLLG afferent neurons to stabilize synaptic contacts.

### Loss of Nsf affects BDNF function

**Figure 7:**
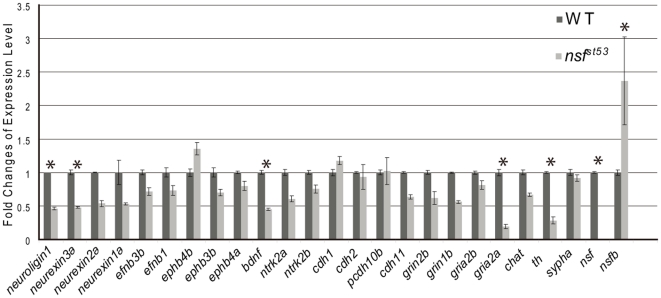
Expression of genes important for synaptic stability is reduced in *nsf^st53^* mutants. QPCR was used to determine the expression levels of genes in brain cDNA from both wild-type and *nsf^st53^* mutant larvae (5 dpf). Gene expression level in wild-type larvae was normalized to 1. Genes indicated with “*” showed 2-fold changes or more with p<0.001.

Many components that promote synaptic stabilization can potentially be mediated by NSF-dependent membrane trafficking [18], [19]. A previous study examining neuropeptide release found an increase of protein level, but a decrease of mRNA level of several neuropeptides in zebrafish *nsf* mutants [8]. Decreased transcript levels are likely due to transcriptional repression of genes encoding proteins that accumulate within the cell body and fail to be secreted. To determine if this were the case for signaling components in hair-cell synapses, we performed real-time quantitative PCR (qPCR) with larval brain tissue to examine the transcript levels for genes that may be important for synaptic stabilization. Among the 22 genes that we analyzed, 5 of them showed a significant repression of more than 2 fold in brain cDNA from *nsf^st53^* mutant larvae. These genes include *neuroligin 1, neurexin 3a, bdnf, gria2a* and *th* (Figure 7). Expression of *ephrin* and *ephrin receptors*, *cadherins* and *synaptophysin* were unchanged or had less than 2-fold changes (Figure 7). The data obtained from qPCR analysis suggest that signaling pathways involving neurexin-neuroligin, BDNF-TrkB, and neurotransmission are altered by Nsf dysfunction, but Eph receptors, Ephrin ligands, and Cadherins are not affected. In addition, a striking reduction was seen in AMPA receptor transcripts (*gria2a*). Interestingly, mutant *nsf^st53^* transcripts were degraded, whereas wild-type *nsfb* transcripts were increased, perhaps in compensation for the loss of *nsf* (Figure 7).

**Figure 8:**
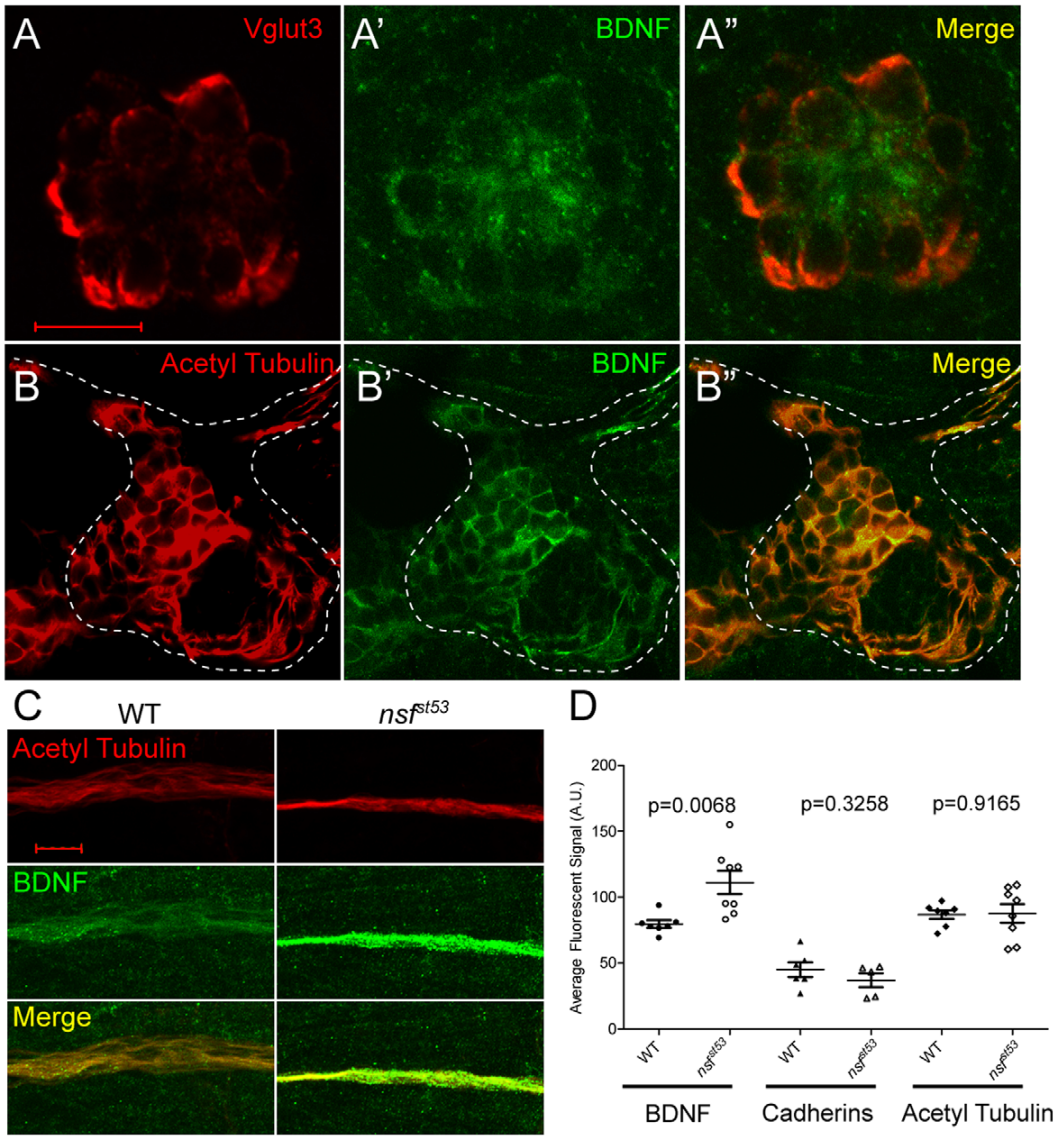
Expression of BDNF in wild-type hair cells and the pLLG, and accumulation of BDNF protein in the lateral line nerve of *nsf^st53^* mutants. **A**, A single optical section of a neuromast from a wild-type zebrafish larva (5 dpf) immunolabeled for Vglut3 (A, red), BDNF (A′, green), and merged in (A″). **B**, A single optical section of a wild-type pLLG, immunolabeled for acetylated Tubulin (B, red), BDNF (B′, green), and merged in (B″). The cell bodies of the pLLG are outlined by dash lines. **C**, Representative z-projections of 6 sections (1 µm each) of acetylated Tubulin (red) and BDNF (green) immunolabeled lateral line nerves in wild-type and *nsf^st53^* mutant larvae. **D**, The average pixel intensity (A.U.) of BDNF (WT, n = 7; *nsf^st53^*, n = 8), PAN-Cadherin (WT, n = 8; *nsf^st53^*, n = 5), and acetylated Tubulin labeling (WT, n = 7; *nsf^st53^*, n = 8) in wild-type control and *nsf^st53^* mutants. Scale bar is 10 µm across all panels.

Of the pathways affected, the BDNF pathway is particularly intriguing. Our data suggest that Nsf function is required in both pre- and postsynaptic cells, and BDNF has been shown to be released from both sides of the synaptic cleft [20]. *bdnf* mRNA and protein have been previously detected in hair cells in zebrafish [21], [22], but BDNF expression in the lateral line ganglion has not been reported. Using an antibody to detect BDNF [21], we found that BDNF colocalized with both a hair-cell marker, Vesicular glutamate transporter 3 (Vglut3), and acetylated Tubulin in the pLLG (Figure 8A-B″). Thus, BDNF is likely to be secreted from both cell types.

If BDNF secretion depends on Nsf activity, we hypothesized that the level of BDNF protein should be elevated in both hair cells and afferent neurons in *nsf^st53^* mutants. Indeed, BDNF immunolabeling was increased in the cell bodies of afferent neurons (data not shown) and their nerve fibers (Figure 8C), suggesting that BDNF- containing vesicles accumulated within the cytoplasm of *nsf^st53^* mutant neurites. Unfortunately, we could not measure changes in the BDNF staining of hair cells because the background staining of the skin interfered with quantification.

However, as a control, we also examined the levels of proteins that are predicted to be unaffected in the *nsf^st53^* mutant based on our qPCR analysis. Consistent with our qPCR results, the fluorescent intensity of two other antibodies against acetylated Tubulin and pan-Cadherins showed no change between *nsf^st53^* mutants and their wild-type siblings (Figure 8C-D). Thus, transcripts such as *cadherin 1, 2*, and *11* that were unaffected in the *nsf^st53^* mutant did not have altered levels of corresponding protein.

**Figure 9:**
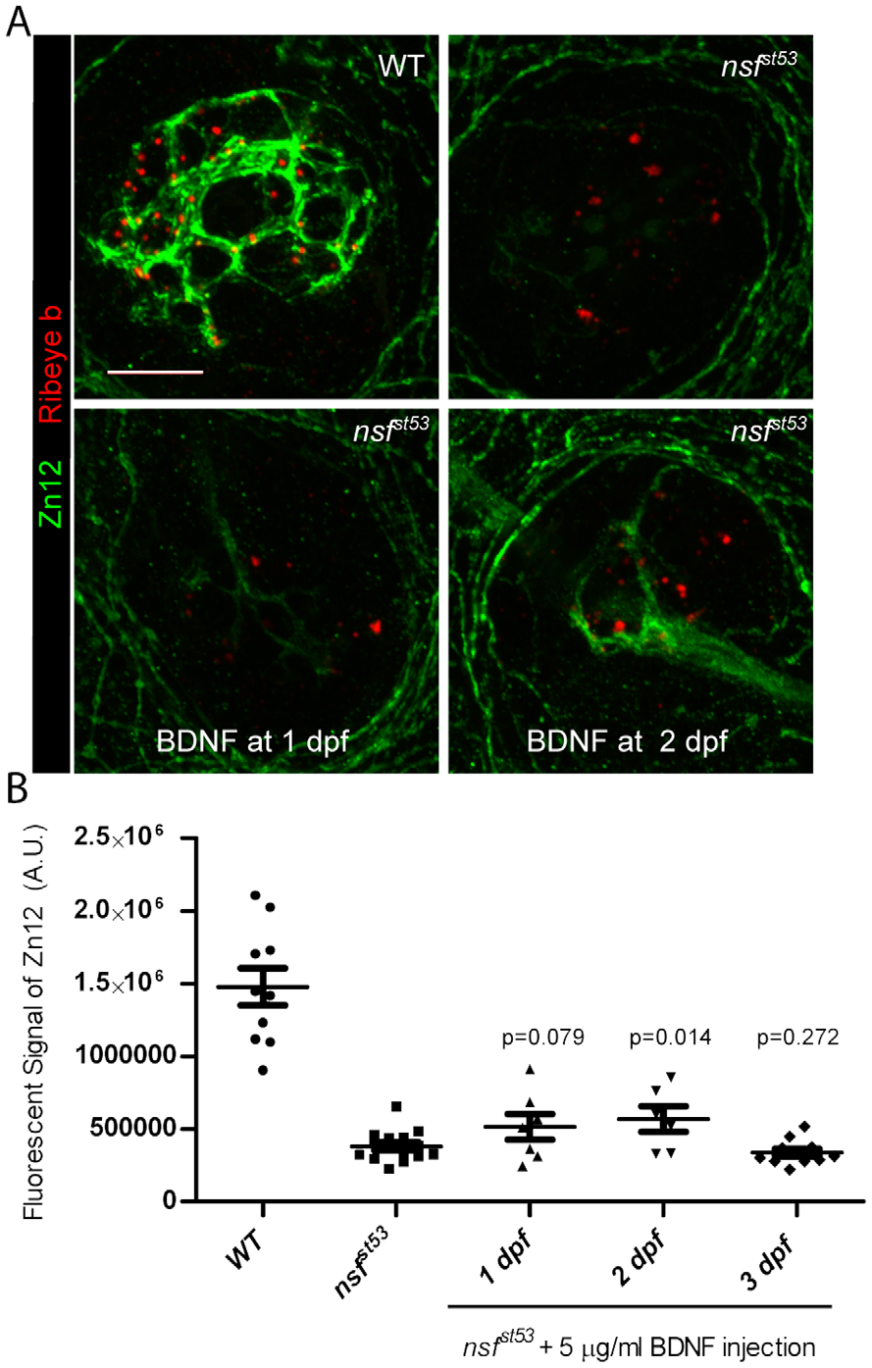
Partial rescue of synaptic contacts in *nsf^st53^* mutants by BDNF injection at 2dpf. **A**, Representative z-projections of single neuromasts (4 dpf) immunolabeled for Zn12 (green) and Ribeye b (red) in wild-type, *nsf^st53^* mutants, and *nsf^st53^*mutants injected with 5 µg/ml BDNF at 1dpf and 2dpf. 10 optical sections, 1 µm each. Scale bar: 10 µm. **B**, Quantification of total pixel intensity (A.U.) of Zn12 labeling in wild-type (1.478e6±128180, n = 10), *nsf^st53^* mutants (382343±26874, n = 15), and mutants with BDNF injections at 1dpf (513236±87981, n = 7), 2dpf (568136±88664, n = 6), 3dpf (339253±25527, n = 11). Statistical tests were between uninjected control and BDNF injected mutants.

Our data suggest that BDNF is acting downstream of Nsf-mediated secretion. In previous studies, injection of recombinant BDNF or incubation of zebrafish larvae in E3 medium containing BDNF protein has been shown to compensate for the loss of endogenous BDNF [23], [24]. We attempted to rescue the de-innervation of lateral line hair cells in *nsf^st53^* mutants by injecting recombinant human BDNF protein into the yolk of *nsf^st53^* mutants at 1 dpf, 2 dpf, and 3 dpf. We then labeled injected fish with Zn12 antibody to visualize afferent fibers (Figure 9A). In *nsf^st53^* mutants injected with BDNF at 2 dpf, we observed a slight increase of afferent fiber labeling compared to uninjected mutants (Figure 9A-B). Injection of BDNF at earlier or later time points did not significantly increase the fluorescent signal of Zn12 labeling (Figure 9A-B), suggesting that there is a critical time window for BDNF to promote afferent synapse stability. Even within this critical period, restoration of innervation was incomplete in our experiments, suggesting that other factors are required downstream of Nsf to prevent retraction of neurites in *nsf^st53^* mutants.

## Discussion

In this study, we examined the pre- and postsynaptic contribution of Nsf in stabilizing zebrafish hair-cell ribbon synapses. Our results indicate that (*i)* mutation of *nsf,* but not *nsfb*, results in retraction of afferent fibers from hair cells, (*ii*) both pre- and postsynaptic Nsf activity are essential for maintaining hair-cell synapses, (*iii*) degeneration of nerve fibers does not result from apoptosis, (*iv*) mutation of *nsf* alters expression of several genes implicated in synaptic stability, and (*v*) BDNF accumulates in *nsf^st53^* mutant afferent fibers, and exogenous BDNF can partially stabilize afferent innervation. Our data suggest a critical role for Nsf in stabilizing synaptic contacts in both the CNS and peripheral nervous system by mediating the expression and secretion of multiple factors important for synaptic maintenance.

### Differing roles of Nsf and Nsfb in zebrafish

The different expression patterns and mutant phenotypes of the *nsf* and *nsfb* genes suggest that these two genes perform non-overlapping functions. *nsf* is expressed mainly in the nervous system, whereas *nsfb* is ubiquitous. In contrast to the neurodegeneration observed in *nsf^st53^* mutants, *nsfb^hi2869Tg^* mutants show an overall progressive degeneration of other tissues at an earlier stage [10]. A similar scenario is seen in *Drosophila,* which also expresses two isoforms of dNSF. *dNSF1* is mainly expressed and functions in the nervous system, while *dNSF2* function is required at the first instar larval stage for mesoderm development [6], [25]. Perhaps most surprising about the *nsf* mutant phenotype in both flies and zebrafish is that early development is not disrupted. The relatively normal development in single mutants may be due to compensation by the unaffected gene. Indeed, we observe increased levels of *nsfb* mRNA in the *nsf^st53^* mutants. However, the *nsfb* gene is unable to compensate for the retraction and degeneration of neurites in either the central or peripheral nervous system of *nsf^st53^* mutants. This suggests that *nsf* plays a specialized role in the maintenance of the synaptic contacts.

### Nsf and neurodegeneration

Our data indicate that neurodegeneration is a prominent phenotype in both CNS or hair-cell glutamatergic synapses in *nsf^st53^* mutants. Interestingly, the motility of *nsf^st53^* mutants is largely normal at 2 dpf compared to their wild-type siblings, but they gradually become paralyzed from 3 dpf to 5 dpf (data not shown). This progressive loss of motility correlates with the timeline of the neurodegenerative phenotypes observed in *nsf^st53^* mutants (Figure 3). In agreement with our observations, neurodegeneration has also been observed in a number of mouse models with defects in SNARE-complex function [2], [26], [27], [28]. The observation of presynaptic disorganization at early stages of neurodegeneration, as well as the crucial function of these proteins in presynaptic vesicle fusion, lends support to the idea that presynaptic dysfunction contributes to neurodegeneration [29]. Our experiments indicate that maintenance of hair-cell synapses requires Nsf function in both pre- and postsynaptic cells. What could account for this difference in pre- versus postsynaptic roles? It is possible that early presynaptic dysfunction in the mouse CNS does not necessarily exclude a postsynaptic contribution to neurodegeneration. The role of NSF in the regulation of GluR2 externalization at post-synaptic membranes is well established [30], [31], [32], and our data clearly demonstrate that *gria2a* AMPA receptor transcripts are decreased in *nsf^st53^* mutants. Unfortunately, we were unable to immunolabel AMPA receptors in zebrafish larvae. However, our results indicate that postsynaptic BDNF accumulates in the neurites of afferent neurons in *nsf^st53^* mutants. Moreover, release of mammalian BDNF is thought to occur at both pre- and postsynaptic sites [20]. Our experiments of pre- versus postsynaptic expression of *nsf* support the notion that release and membrane trafficking in both cell types is required for maintenance of hair-cell synapses.

### Myelination and neurodegeneration

Nsf has been shown to be indispensible for the development of myelination and nodes of Ranvier in zebrafish larvae [9]. Here we revealed the role of Nsf in preventing neurodegeneration. Because of the long-known neuroprotective role of glial cells and myelin protein [33], the loss of innervation could be due to demyelination in *nsf^st53^*. Indeed, we observed that Myelin basic protein and acetylated Tubulin were present in the myelin sheath and lateral line nerves (respectively) at 3 dpf, but the levels of both were dramatically reduced from 3 dpf to 5 dpf in *nsf^st53^* mutants (Figure S4). Since the loss of myelination and innervation occurs coincidentally in *nsf^st53^* lateral line nerves, it is difficult to determine a causal relationship with respect to degeneration. The production of myelin is in part, however, dependent on neuronal signaling. For example, neuregulins are key signals secreted by neurons for Schwann cell development and myelination [34], and our data indicate that zebrafish Nsf is required for release of neurotrophic factors, such as BDNF. It is likely that Nsf promotes myelination through release of gliatrophic factors from lateral line neurons. In contrast to lateral line neurons, the glutamatergic neurons of the CNS that we examined do not have a myelin sheath (Figure S5). The neuroprotective role of Nsf can therefore be independent of myelination.

### Nsf function in hair cells

An interesting finding of this study is that Nsf in hair cells is required for synaptic stability in hair cells. Despite some differences in exocytic machinery at ribbon synapses, many core proteins of the SNARE-complex that interact with NSF have been detected in ribbon synapses isolated from chicken hair cells and in mouse cochlea hair cells [35], [36]. The presence of SNARE proteins is indicative of a role in membrane fusion, however, botulinum neurotoxins that normally cleave SNAP25 and Syntaxins in conventional synapses have no effect on synaptic transmission in mouse inner hair cells [37]. Our results do not address whether zebrafish Nsf is required for synaptic transmission, but rather indicate that in the absence of Nsf activity, afferent neurons fail to maintain stable contacts with hair cells. Based on the reduction in transcripts for synaptic proteins that have been implicated in maintenance, and the internal accumulation of BDNF, Nsf appears to participate in trafficking components required for synaptic stability. Our results indicate that Nsf-mediated activity is required on both sides of the cleft and we speculate that core components of the SNARE-complex are likely to be involved in this process.

### Factors acting downstream of Nsf-mediated vesicle fusion

One of the most widely studied signaling molecules released from both pre- and postsynapses is BDNF. Release of BDNF triggers both pre- and postsynaptic signaling events [38], [39], [40], and is important for synaptic maintenance in the brain [41], [42]. Loss of BDNF in zebrafish larvae affects early neuronal development and causes a more severe phenotype than *nsf^st53^* mutants [23], [43]. Blockade of synaptic transmission is also known to reduce neurotrophic factor secretion [44], thus it is possible that the neurodegeneration in *nsf^st53^* mutants is due to lack of trophic support. Indeed, the presence of higher levels of BDNF in *nsf^st53^* afferent neurons suggests that trafficking and secretion of BDNF is reduced at the hair-cell synapse. BDNF injection or incubation has been successfully used to rescue neuronal growth in zebrafish larvae [23], [24], however, we observed only a partial stabilization of afferent innervation of hair cells in *nsf^st53^* mutants that received an injection of recombinant BDNF. The partial rescue suggests that BDNF alone is insufficient and may simply delay but not rescue neurodegeneration. Similar results have been observed in mouse *Munc-18-1* mutants; the neurodegeneration phenotype was delayed but not rescued by application of BDNF and insulin [45]. Partial rescue with BDNF can be explained in several ways. First, BDNF can stimulate cell survival [46], [47] and injection of BDNF into *nsf^st53^* mutants may have delayed the retraction of afferent neurites. Second, exogenous BDNF may not be able to reach the synaptic cleft efficiently; hence full rescue of the phenotype is not possible even if BDNF were sufficient to stabilize synaptic contacts. Third, it is possible that the trafficking of TrkB receptors is affected in *nsf^st53^* mutants. Lastly, BDNF is likely to be one of many factors secreted at hair-cell synapses. Indeed, our expression data indicates that the BDNF pathway is potentially one of multiple signaling or adhesion complexes that may be required for stabilization of synaptic contacts. Further investigation may reveal which of these factors mediates synaptogenesis in hair cells, but regardless of the downstream signaling events, our data suggest that both sides of the hair-cell synaptic cleft require Nsf for long-term maintenance of synapses.

## Materials and Methods

### Zebrafish strains and husbandry

Adult zebrafish stains were maintained as previously described [48]. All lines used in this study were maintained in a Tübingen or Top Long Fin background. *TgBAC(neurod:EGFP)nl1* transgenic fish were previously described [11]; the *nsf^st53^* [9] and *nsfb^hi2869Tg^* allele [10] were obtained from the Zebrafish International Resource Center.

### Transgenic fish

Stable transgenic lines were generated as described previously [49]. The promoter sequence of *myo6b* gene (-6myo6b), described previously [11], was cloned into a 5-prime entry (p5E) vector in the Tol2 kit vector #381 p5E-MCS [49]. To clone the promoter sequence of *neurod* gene, the following primers were used to amplify and insert an approximate 5 kb fragment (-5neurod) into the same p5E vector: Forward primer with Fse I site: GGCCGGCCCGGCATCAAACCGCCTCG AGAG, Reverse primer with Asc I site: GGCGCGCCGTCGGAACTCTGCAAAGC GATAAAGC.

### Phylogenetic analysis

Protein sequences of Nsf from different species were obtained from UCSC genome browser (http://genome.ucsc.edu/). They are yeast NSF (YBR080C), nematode NSF (NP_00107660), fly dNSF1 (CG1618-RA) and dNSF2 (CG33101-RA), zebrafish Nsf (NP_001037793) and Nsfb (NP_001019625), mouse NSF (NP_032766), and human NSF (NP_006169). A phylogenetic tree was generated by ClustalW2, statistically evaluated by Bootstrap for 1000 times.

### Immunofluorescent staining

Whole-mount immunostaining was performed according to a previous report with minor changes [13]. Briefly, fish larvae were fixed at 4°C for 4 hours with 4% paraformaldehyde, 4% sucrose, and 0.01% Tween-20 in PBS solution. After rinsing twice with 0.25% Tween-20, and 1% DMSO in PBS (PBSDT), fixed embryos were permeabilized by exposure to 100% acetone at −20°C and then washed with H_2_O and PBSDT. Embryos were blocked with 6% goat serum, 3% bovine serum albumin in PBSDT for 1 hour, and then followed by incubation with primary antibody at 4°C overnight. After rinsing with PBSDT for 2 hours, samples were stained with a secondary antibody at 4°C overnight or 5 hours at room temperature. Primary antibodies used in this study are listed in Table 1. Alexa Fluor-conjugated secondary antibodies (Invitrogen) were used at 1∶1500.

**Table 1:** List of antibodies.

Antibody	Dilution	Suppliers	References
NSF	1∶50	Cell Signaling #3924	
Ribeye b	1∶4000	Generated by Openbiosystems	[13]
Zn12	1∶500	ZIRC, Zn-12	[12]
MAGUK	1∶500	Neuromab, #73-029	[13]
Vglut3	1∶1000	Generated by Proteintech	[11]
Vglut1	1∶1000	Generated by Proteintech	[50]
Casp3	1∶250	Cell signaling #9661	[51]
BDNF	1∶100	Santa Crutz, sc-65513	[21]
Cadherins	1∶500	Sigma, C3678	[52]
MBP	1∶50	Generated by Talbot lab	[9]
Acetylated-Tubulin	1∶1500	Sigma, T6793	[12]
GFP	1∶500	Aves lab, #GFP-1020	[53]

### Confocal microscopy and quantification

Specimens were mounted in Elvanol mounting media and dried overnight. Confocal imaging and data analysis procedure was described previously [13] with the following changes. Z-stack images were acquired using a Zeiss LSM 700 confocal microscope with either 10X or 60X oil lens using Zen software and the pinhole was set to 1 auto unit. Filters were set at default settings for Alex 488, 568 or 647 fluorescent signals. Z-stack images were transformed into Tiff images with Image J (NIH, Bethesda, MD, USA) before being analyzed by Metamorph (Molecular Devices, Sunnyvale, CA, USA). Using the maximal projection of each image, individual neuromasts were selected manually and the background, defined as the average intensity of the whole image, was deducted before using the integrated morphometry analysis function for quantification of the fluorescent intensity of antibody labeling or the number of punctae. Individual neuromasts were selected based on the fluorescent signals of Ribeye b staining, which specifically labels the presynaptic ribbon synapses in hair cells. A punctum was defined as containing ≥20 pixels with three-fold intensity above background. To quantify BDNF, Pan-Cadherins, Acetylated Tubulin and MBP staining in LLN, the LLN was selected manually on a maximum projection image based on the fluorescent signal of the Acetylated Tubulin antibody. Only positively labeled regions were used for calculating the average fluorescent signals in Fig 8. For all other figures, the total intensity of antibody labeling is shown. A region before the first melanophore was selected for all images of LLNs. All quantities in figures are presented as mean ± standard error. 2-tailed unpaired student t test was used to compare two groups of data.

### Quantification of apoptosis and cell numbers

A confocal Z-stack with 10 slices was taken from selected regions. For analysis of apoptosis, one digital section image with the most apoptotic cells was picked from each Z-stack. Apoptotic and normal cells were counted in every image. To count cell numbers in each image, the digital section with the most cell numbers was chosen from each Z-stack scanning. Cells were counted based on the GFP fluorescence from *TgBAC(neurod:EGFP)nl1* transgenic fish and/or DAPI staining in each image. Numbers were then analyzed using Microsoft Excel (Microsoft, WA) and graphs were made using Prism (GraphPad Software, CA).

### RT-PCR and qPCR

5 days post fertilization (dpf) zebrafish larvae or adult fish were anaesthetized in MESAB/Tricaine solution, and then tissues from adult fish or larvae were dissected and immediately put into RNAlater (Applied Biosystems/Ambion). Total RNA was extracted from wild-type or *nsf* mutants using the RNeasy mini kit (Qiagen). 5 µg RNA was then reverse transcribed using the EcoDry premixes (Clontech, # 639541). For qPCR purpose, 0.2 µl cDNA in 10 µl SYBR green mixture in 384-well plates was used for each qPCR reaction on an Applied Biosystems 7900 HT real-time PCR machine. The RNA level for each gene was first calculated from a cDNA standard curve and then normalized to *actin* RNA. To perform RT-PCR, 0.2 µl cDNA was used in a 20 µl PCR reaction.

## Supporting Information

Figure S1 **Glutamatergic synapses in the cerebellum.** Magnified images of the boxed region in Figure 4A′ showed fluorescent signals from Vglut1 (red) and MAGUK (green) antibodies in Purkinje cells of the cerebellar region. Although MAGUK antibody labels both cell body and postsynaptic density in these neurons, it is possible to observe juxtaposition of MAGUK densities next to Vglut1 labeled presynaptic terminals (arrow heads). Scale bar is 10 µm.
(TIF)

Figure S2 **Quantification of GFP fluorescence in** ***Tg(-6myo6b:nsf-GFP)vo1*** **and** ***Tg(-6myo6b:nsf-GFP)vo2*** **lines.** Anti-GFP antibody was used to stain Nsf-GFP fusion proteins in *Tg(-6myo6b:nsf-GFP)*/*nsf^st53^* mutants. The fluorescent intensity in the neuromasts of *Tg(-6myo6b:nsf-GFP)vo2* (2.353e6±293299, n = 17) and *Tg(-6myo6b:nsf-GFP)vo1* (971656±215772, n = 10) lines are significantly different.
(TIF)

Figure S3 **Rescue of afferent innervation by double transgenic expression of Nsf-GFP in** ***nsf^st53^*** **mutants.** **A-E**″, Top-down views of the first lateral line neuromast (5 dpf) from wild-type (**A**), *nsf^st53^* mutants (**B**), and *nsf^st53^* mutants with Nsf-GFP expressed in hair cells (**C**), pLLG (**D**), or in both hair cells and pLLG (**E**). Shown is immunolabeling with antibodies against GFP (light blue), Zn12 (green), and Ribeye b (red). Scale bar: 10 µm. **F**, The total intensity of Zn12 antibody labeling per neuromast was quantified in wild-type (2.113e6±259343, n = 6),*nsf^st53^* mutants (133379±80303, n = 4), and *nsf^st53^* mutants with Nsf-GFP rescued in hair cells (559662±104829, n = 13), pLLG (684537±75475, n = 12), or both hair cells and pLLG (1.659e6±136058, n = 12).The p-values were generated comparing the data from the *nsf^st53^* mutant to each transgenic mutant line.
(TIF)

Figure S4 **Decreased labeling of acetylated Tubulin and Myelin Basic Protein in** ***nsf^st53^*** **mutants.** **A,** Antibodies against acetylated Tubulin were used to label lateral line nerves in wild-type and *nsf^st53^* mutant larvae at 3, 4 and 5 dpf. **B**, Anti-Myelin Basic Protein antibody labeled the myelin sheath of the lateral line nerve in both wild-type and *nsf^st53^* mutants (3 to 5 dpf). **C**, The fluorescent intensity of acetylated Tubulin labeling increased over time in wild-type larvae (3 dpf, 1523307±233786; 4dpf, 1445077±252144; 5 dpf, 2170483±434340), but decreased in *nsf^st53^* mutants (3 dpf, 1201135±110419; 4dpf, 770785±58768; 5 dpf, 264018±35511). **D**, The fluorescent labeling of Myelin Basic Protein displayed a dramatic increase in wild-type (3 dpf, 1155025±158382; 4dpf, 1746149±232916; 5 dpf, 3501120±670992), but significantly decreased in *nsf^st53^* mutants (3 dpf, 860546±74798; 4dpf, 538577±85907; 5 dpf, 212506±24043). Scale bar: 10 µm; z-projection of 5 confocal planes (1 µm each).
(TIF)

Figure S5 **Myelin Basic Protein expresses in peripheral nerves, but not in the CNS.** **A-A‴**, A representative top-down projection of a zebrafish brain at 5 dpf labeled by GFP in the *TgBAC(neurod:EGFP)nl1* background (**A**, light blue), antibodies against acetylated Tublin (Acetyl Tub, **A**′, green) and Myelin Basic Protein (MBP, **A**″, red). MBP protein was not detected in the CNS. Scale bar: 100 µm. **B-B‴**, Close up of the boxed region in panel **A‴**. Although MBP fluorescence was associated with nerve fibers from the anterior lateral line ganglion (arrow head), there was no labeling of MBP in nerve fibers in the cerebellar region (highlighted by GFP expression). Scale bar: 10 µm.
(TIF)
