# Supplementary figures and images for: Both Pre- and Postsynaptic Activity of Nsf Prevents Degeneration of Hair-Cell Synapses
Source: PLoS One. 2011 Nov 3;6(11):e27146. doi: 10.1371/journal.pone.0027146 (PMC3207842; doi:10.1371/journal.pone.0027146)

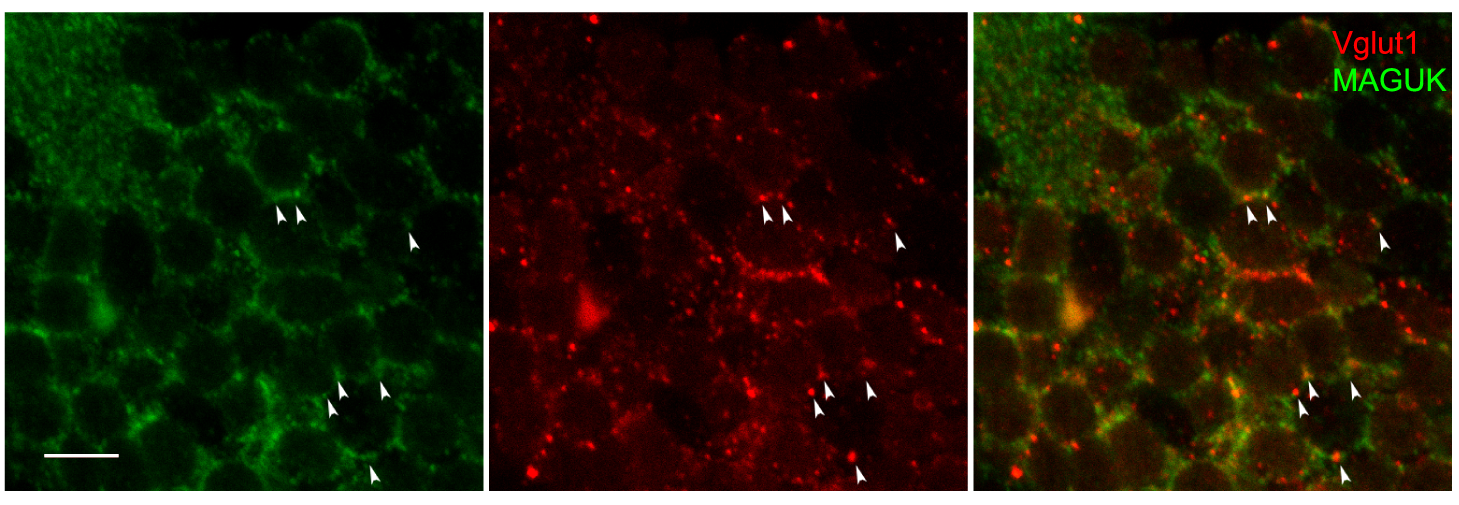

Supplement: Figure S1 — Glutamatergic synapses in the cerebellum. Magnified images of the boxed region in Figure 4A′ showed fluorescent signals from Vglut1 (red) and MAGUK (green) antibodies in Purkinje cells of the cerebellar region. Although MAGUK antibody labels both cell body and postsynaptic density in these neurons, it is possible to observe juxtaposition of MAGUK densities next to Vglut1 labeled presynaptic terminals (arrow heads). Scale bar is 10 µm. (TIF) [file pone.0027146.s001.tif]

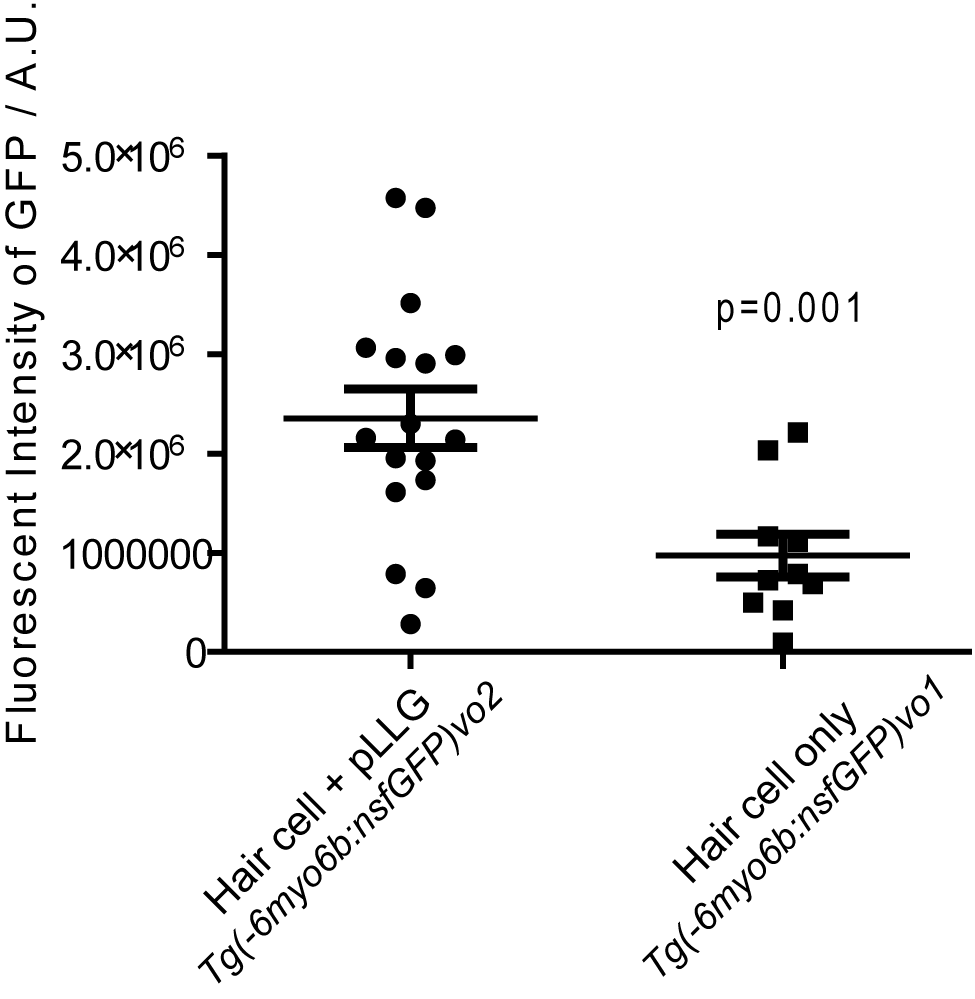

Supplement: Figure S2 — Quantification of GFP fluorescence in Tg(-6myo6b:nsf-GFP)vo1 and Tg(-6myo6b:nsf-GFP)vo2 lines. Anti-GFP antibody was used to stain Nsf-GFP fusion proteins in Tg(-6myo6b:nsf-GFP)/nsfst53 mutants. The fluorescent intensity in the neuromasts of Tg(-6myo6b:nsf-GFP)vo2 (2.353e6±293299, n = 17) and Tg(-6myo6b:nsf-GFP)vo1 (971656±215772, n = 10) lines are significantly different. (TIF) [file pone.0027146.s002.tif]

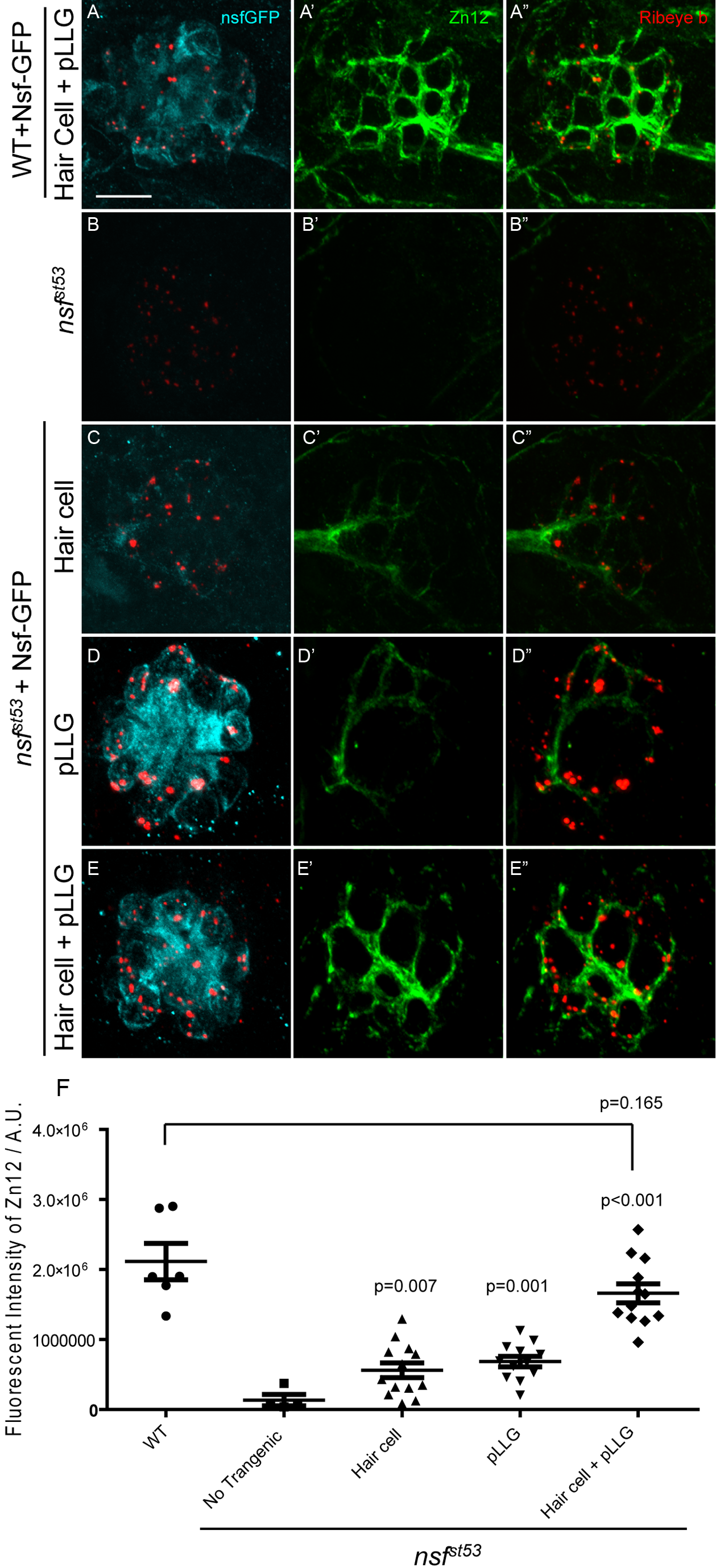

Supplement: Figure S3 — Rescue of afferent innervation by double transgenic expression of Nsf-GFP in nsfst53 mutants. A-E″, Top-down views of the first lateral line neuromast (5 dpf) from wild-type (A), nsfst53 mutants (B), and nsfst53 mutants with Nsf-GFP expressed in hair cells (C), pLLG (D), or in both hair cells and pLLG (E). Shown is immunolabeling with antibodies against GFP (light blue), Zn12 (green), and Ribeye b (red). Scale bar: 10 µm. F, The total intensity of Zn12 antibody labeling per neuromast was quantified in wild-type (2.113e6±259343, n = 6),nsfst53 mutants (133379±80303, n = 4), and nsfst53 mutants with Nsf-GFP rescued in hair cells (559662±104829, n = 13), pLLG (684537±75475, n = 12), or both hair cells and pLLG (1.659e6±136058, n = 12).The p-values were generated comparing the data from the nsfst53 mutant to each transgenic mutant line. (TIF) [file pone.0027146.s003.tif]

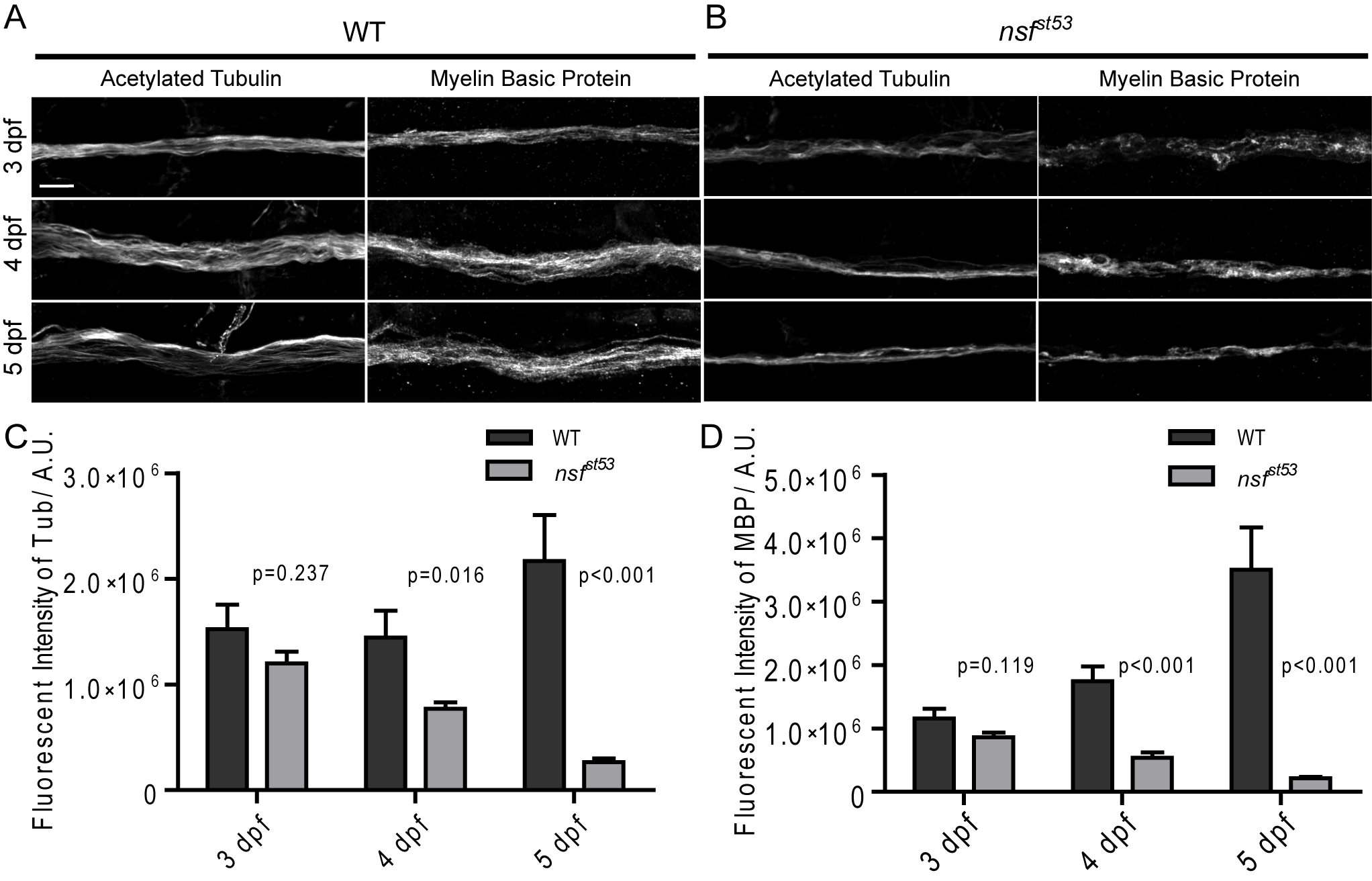

Supplement: Figure S4 — Decreased labeling of acetylated Tubulin and Myelin Basic Protein in nsfst53 mutants. A, Antibodies against acetylated Tubulin were used to label lateral line nerves in wild-type and nsfst53 mutant larvae at 3, 4 and 5 dpf. B, Anti-Myelin Basic Protein antibody labeled the myelin sheath of the lateral line nerve in both wild-type and nsfst53 mutants (3 to 5 dpf). C, The fluorescent intensity of acetylated Tubulin labeling increased over time in wild-type larvae (3 dpf, 1523307±233786; 4dpf, 1445077±252144; 5 dpf, 2170483±434340), but decreased in nsfst53 mutants (3 dpf, 1201135±110419; 4dpf, 770785±58768; 5 dpf, 264018±35511). D, The fluorescent labeling of Myelin Basic Protein displayed a dramatic increase in wild-type (3 dpf, 1155025±158382; 4dpf, 1746149±232916; 5 dpf, 3501120±670992), but significantly decreased in nsfst53 mutants (3 dpf, 860546±74798; 4dpf, 538577±85907; 5 dpf, 212506±24043). Scale bar: 10 µm; z-projection of 5 confocal planes (1 µm each). (TIF) [file pone.0027146.s004.tif]

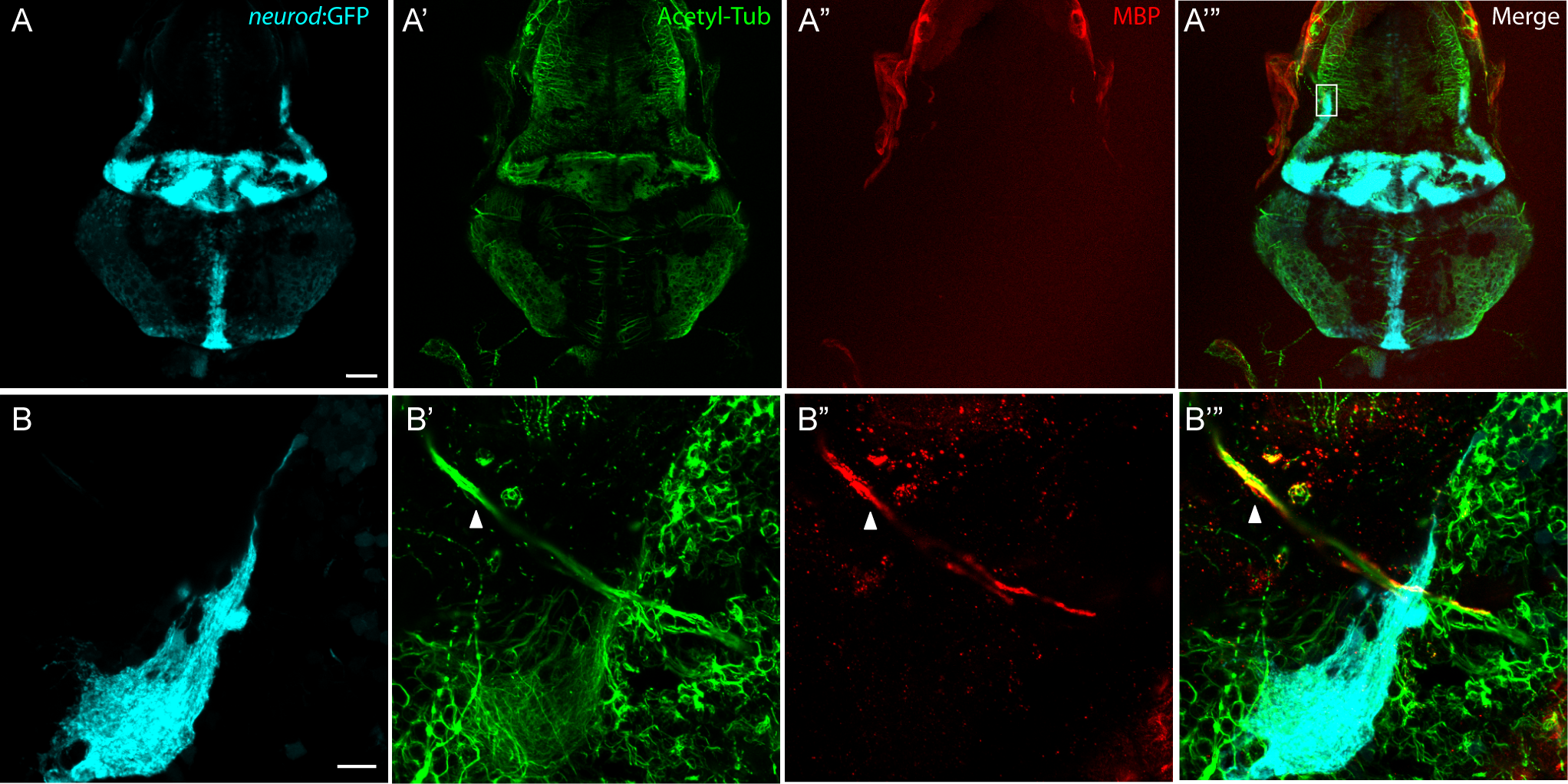

Supplement: Figure S5 — Myelin Basic Protein expresses in peripheral nerves, but not in the CNS. A-A‴, A representative top-down projection of a zebrafish brain at 5 dpf labeled by GFP in the TgBAC(neurod:EGFP)nl1 background (A, light blue), antibodies against acetylated Tublin (Acetyl Tub, A′, green) and Myelin Basic Protein (MBP, A″, red). MBP protein was not detected in the CNS. Scale bar: 100 µm. B-B‴, Close up of the boxed region in panel A‴. Although MBP fluorescence was associated with nerve fibers from the anterior lateral line ganglion (arrow head), there was no labeling of MBP in nerve fibers in the cerebellar region (highlighted by GFP expression). Scale bar: 10 µm. (TIF) [file pone.0027146.s005.tif]
